# Chronic respiratory morbidity and wheeze after preterm birth: a narrative review

**DOI:** 10.3389/fped.2026.1880728

**Published:** 2026-08-27

**Authors:** A. Jenkinson, S. Ahmed, A. Gupta, A. Greenough

**Affiliations:** 1 Department of Women and Children’s Health, School of Life Course and Population Sciences, Faculty of Life Science and Medicine, King’s College London, London, United Kingdom; 2 Department of Neonatology, King’s College Hospital, London, United Kingdom; 3 Department of Paediatric Respiratory Medicine, King’s College Hospital, London, United Kingdom

**Keywords:** neonatolgy, preterm (birth), pulmonology, risk factors, wheeze

## Abstract

Preterm birth, defined as birth before 37 weeks of gestation, affects approximately 10% of pregnancies worldwide. Premature birth disrupts normal lung development with those born at earlier gestations most affected. Individuals born preterm are at increased risk of lifelong respiratory morbidity, including recurrent wheeze. As a consequence, affected individuals are often given an incorrect diagnosis of asthma. Asthma is considered a clinical description and is defined as a clinical syndrome characterised by wheeze, breathlessness, and chest tightness, sometimes accompanied by excess cough. This narrative review reports wheeze following preterm birth, considers prematurity-associated lung disease as an alternative diagnosis to asthma and discusses early life exposures as modifiable factors. Furthermore, whether prediction of those at highest risk is possible and what is the optimum management and follow up for affected individuals is discussed. Preterm birth is associated with an approximately double the risk of wheezing in childhood and is particularly common in those born extremely prematurely. While type two (T2) asthma is the predominant asthma phenotype in the general paediatric population, non-T2 mechanisms may be more relevant in those born preterm and hence why they are often unresponsive to standard asthma regimens. Potentially modifiable risk factors are antenatal corticosteroids, *in utero* growth retardation, chorioamnionitis, maternal antenatal smoking, breast feeding, neonatal antibiotic exposure, aspiration lung disease, viral infections, pollution, the microbiome and socio-economic factors. Prematurely born individuals have a lower lung function trajectory than those born at term, predictors of those at higher risk may be possible using either a definition of BPD that emphasises the respiratory support needed at 36 weeks PMA or cluster analysis using clinical data. The heavy burden of chronic respiratory morbidity in this population warrants their lifelong follow-up and a personalised approach to their management.

## 1 Introduction

Preterm birth (birth before 37 weeks of gestation) affects approximately 10% of live births worldwide (1). Despite improvements in care, the global prevalence of preterm birth remains high (2). In 2021, the overall rate of preterm birth in the United Kingdom (UK) increased to 7.5% (3). In the United States preterm birth exceeds 10% of live births (4). Wheeze at follow-up is common in this population and as a result preterm individuals often receive an asthma diagnosis (5). Hence, the prevalence of asthma among preterm infants is reported to be as high as 32.7% at two years (6), but the diagnosis of asthma in preterm-born individuals is increasingly questioned (7). Unlike classical paediatric asthma, which is typically characterised by reversible airway obstruction and type 2 (T2) inflammation, respiratory problems following preterm birth arises in the context of disrupted lung development and hence may reflect a different pathophysiology (8). Advances in obstetric and neonatal care have resulted in improved survival to adulthood (9, 10) and the threshold of viability now includes infants born from 22 weeks of gestation (11–13). As a consequence, there is an increasing population of preterm survivors and with their high burden of respiratory morbidity, there is a need to better understand the influencing factors. The aims of this narrative review then are to report wheeze following preterm birth, consider prematurity-associated lung disease as an alternative diagnosis to asthma and discuss early life exposures as modifiable factors. Furthermore, we will discuss whether prediction of those at highest risk is possible and what is the optimum management and follow up for affected individuals.

## 2 What is “asthma”

The Lancet commission proposed that asthma is a clinical syndrome of wheeze, shortness of breath, chest tightness and sometimes increased cough (14). This makes no assumptions about underlying pathology. “Asthma” is thus a description, not a diagnosis. The next step is to determine the specific asthma phenotype present in the patient, for example, fixed and variable airflow obstruction, airway inflammation, and airway infection. Management should then be directed towards any identifiable and treatable components (15). The Lancet definition provides a pragmatic, symptom-based approach to diagnosing asthma, enabling diagnosis prior to the age at which reliable, volitional lung function testing is feasible, and offering utility in low-resource settings where spirometry is unavailable. However, a key limitation is the potential for overdiagnosis of asthma in children with wheeze, particularly in populations with underlying structural lung abnormalities.

Asthma, as defined by GINA 2026, is a heterogeneous disease characterised by chronic airway inflammation, presenting with variable respiratory symptoms including wheeze, breathlessness, chest tightness, and cough and associated with variable expiratory airflow limitation (16). In children, particularly in preschool age groups, the diagnosis is primarily clinical, based on recurrent symptom patterns, variability over time, response to treatment, and exclusion of alternative diagnoses. The ERS/ATS defines asthma as symptoms such as wheeze, shortness of breath, chest tightness and cough together with variable airflow limitation on spirometric assessment that is at least partly reversible either spontaneously or with treatment (17, 18). The ERS/ATS clinical practice guidelines emphasise the need for an evidence-based approach for diagnosis of asthma in childhood with a strong emphasis on phenotypes (17, 18).

This move away from a single unifying diagnosis towards mechanism-based sub-classification is not unique to asthma. The 2023 Global Initiative for Chronic Obstructive Lung Disease (GOLD) COPD report introduced a taxonomy of aetiological “etiotypes,” including COPD-D, due to abnormal lung development, which explicitly incorporates preterm birth and early-life lung injury, alongside genetic, infective, and environmentally driven etiotypes (197). This framework was proposed precisely because a single clinical label (“COPD”) was found to obscure distinct underlying causes requiring different management and prognostic counselling. The wheeze and reduced lung function seen after preterm birth arguably represent a paediatric analogue of this developmental etiotype: a syndrome that resembles asthma clinically but arises from disrupted lung development rather than T2 airway inflammation, and for which an aetiology- and trait-based approach rather than an asthma label is likely to be more useful for both management and research.

## 3 Wheeze and asthma after preterm birth

In a meta-analysis of 30 studies including more than 1,500,000 children, preterm birth compared to birth at term was associated with an increased risk of childhood wheezing disorders [13.7% vs. 8.3%; odds ratio [OR] 1.71, 95% confidence interval [95% CI] 1.57–1.87] (19). Outcome definitions varied amongst the included studies from a symptoms based, prescription based and hospital admission based definition of asthma. In a Boston cohort of approximately 2,500 children (including both preterm and term-born participants) assessed at ages 0–5 and 6–9 years, preterm birth was associated with an increased risk of asthma compared with term birth [adjusted odds ratios (AOR) 1.8–2.9], with a “dose-response” relationship whereby early preterm birth (22–31 weeks) had the highest risk (AORs up to 6.2) and late preterm birth (32–36 weeks) a more modest increase (1.5–2.5) (20). The study by He et al. reported symptoms based and prescription based definitions of asthma. A national cohort study from Sweden following approximately 4,000,000 individuals from birth to adulthood demonstrated that preterm birth was associated with an increased asthma risk beyond childhood defined as per the International Classification of Diseases classifications of asthma (5). The increased risk of asthma as gestational age decreased was not explained by familial, genetic or early life environmental factors. This effect was observed across all age groups with adjusted hazard ratio [AHR] of 3.01 (95% CI: 2.88 to 3.15) for extremely preterm (22–27 weeks), 1.76 (95% CI: 1.72 to 1.79) for very or moderately preterm (28–33 weeks) and 1.31 (95% CI: 1.29 to 1.32) for late preterm (34–36 weeks) (5). Preterm birth was consistently associated with increased asthma risk across birth decades including in individuals born in the 2000s (AHR: 1.59, 95% CI: 1.56–1.62), compared with individuals born in the 1980s (AHR: 1.56, 95% CI: 1.49–1.63). This indicates that the association also occurs in the post-surfactant era despite advances in neonatal and perinatal care. This registry data reported physician diagnosed asthma and did not have pulmonary function data available (5). A national cohort study from Korea identified newborns born in the post-surfactant era (2008–2014) who were followed up to the age of six years of age (21). The study cohort of over two million individuals were divided into three groups: 3,518 (0.2%) were defined as extremely preterm (EP, less than 28 weeks gestation), 82,579 (3.7%) were defined as “other preterm” (OP between 28 and 36 weeks gestation) and the remaining infants were defined as full term (FT greater than 36 weeks gestation). The incidence of asthma was the highest in the EP group, followed by the OP group, and then the FT group (32.0% vs. 21.8% vs. 17.2%, *p* < 0.001). BPD emerged as a significant risk factor for both severe and early-onset asthma (OR: 1.36, 95% CI: 1.21–1.37) for severe asthma which was defined by hospital admission.

The most common asthma phenotype in children is T2-high characterised by eosinophilic airway inflammation (22–24). There is emerging evidence that a T2-low/nonatopic “asthma” phenotype may be prevalent in the preterm population (25–29). Increased pulmonary inflammatory markers and neutrophil counts in the blood and airways have been reported in some studies, suggesting that the inflammation seen in the preterm population may be neutrophilic or non atopic rather than eosinophilic (25, 27, 30). Ex-preterm adults have shown reduced likelihood of positive skin prick testing and lower allergen-specific IgE compared with term controls (25). Fractional exhaled nitric oxide (a marker typically elevated in eosinophilic asthma) has not been shown to be elevated in preterm-born cohorts (27). Consistent with this, induced sputum from school-age children born before 32 weeks of gestation has demonstrated significantly higher neutrophil counts and IL-8 concentrations compared with term controls, providing more direct evidence of a neutrophil-predominant inflammatory process (30). Neutrophilic inflammatory phenotypes in asthma are less likely to respond to first line treatment approaches (31); neutrophilic inflammation may be responsible for 50% of corticosteroid resistant asthma (32). Those findings highlight the need to distinguish asthma phenotypes associated with prematurity as the application of conventional asthma frameworks may lead to misclassification and inappropriate management in that population.

## 4 Prematurity-associated lung disease and its phenotypes

Phenotypes in the long term follow up of preterm infants based on spirometric assessments have been described: prematurity-associated obstructive lung disease (POLD), prematurity-associated preserved ratio of impaired spirometry (pPRISm) and prematurity-associated dysanapsis (pDysanapsis) (33). Wheeze as a symptom during their lifetime was reported to be between 72%–79% in POLD and pDysanapsis as opposed to pPRISm phenotypes where it was reported as 55% which was more in line with the preterm “normal spirometry” cohort (33). Comparison to a term cohort, however, was not made. The same group reported phenotypic variations in bronchodilator reversibility of the POLD phenotype with a proportion of these infants having a fixed airway obstruction and a portion having some evidence of bronchodilator reversibility (33). There is, however, lack of evidence as to whether individuals may present with different spirometric phenotypes at different timepoints.

Simpson et al. proposed a pragmatic “hub and spoke” model for the characterisation of prematurity-associated lung disease (PLD), in which a central disease process (“hub”), arising from disrupted lung development and early-life injury, is linked to multiple interacting domains (“spokes”) including structural abnormalities, physiological impairment, inflammatory pathways and clinical symptoms (34). This model recognises that these domains may coexist and vary between individuals, resulting in heterogeneous phenotypic presentations. In contrast to spirometry-based classifications, which primarily define PLD in terms of airflow obstruction, the hub and spoke model emphasises that respiratory symptoms and asthma-like features arise from a complex interplay of developmental, inflammatory and functional risk factors (34, 35).

## 5 Risk factors as modifiers

### 5.1 Antenatal corticosteroids

Corticosteroids are frequently administered during the antenatal period to stimulate fetal lung maturation and thus help mitigate the complications associated with preterm births (36). A Cochrane Review, which included 30 studies, evaluating the effectiveness of corticosteroid therapy in women at risk of preterm birth showed that the treatment reduced the risk of respiratory distress syndrome (RDS) (average RR: 0.66, 95% CI: 0.56 to 0.77) and the requirement for ventilatory support (RR: 0.68, 95% CI: 0.56 to 0.84). Furthermore, a recent systematic review of six randomised control trials in late preterm infants (defined as gestations between 34 0/7 and 36 6/7 weeks) found that exposure to antenatal corticosteroids (ACS) was associated with decreased use of CPAP [Relative Risk (RR) 0.78, 95% CI: 0.65–0.94, *p* = 0.007] and surfactant (RR: 0.61, 95% CI: 0.38–0.99, *p* = 0.04) (37). Those observed effects are mediated by the multiple actions of corticosteroids on the neonatal lungs including, alterations in gene expression, promotion of surfactant production and improving lung compliance (38, 39). Gender also appears to influence outcomes. A large cohort study of 11,714 preterm infants assessing the effect of ACS on neonatal morbidity demonstrated that females had a lower rate of bronchopulmonary dysplasia (BPD) (61.6 vs. 68.2%, *p* = <0.001) and shorter length of time on mechanical ventilation [9 (3–26) vs. 13 (4–29) days, *p* = <0.001] compared to males (40). This trend was further observed in a cohort study of 319 school aged children who were born prematurely which showed that the lung function in males was poorer than in females (41). Evidence regarding the long-term respiratory outcomes of ACS, particularly wheeze and asthma, remains limited. A recent prospective study of 1,218 premature infants, however, found that antenatal administration of betamethasone was associated with a reduced incidence of wheeze in childhood (42). The optimal dosing regimen for antenatal corticosteroids is a single course administered greater than 24 h and less than seven days before birth (43). While multiple courses have been demonstrated to have potential adverse childhood neurodevelopment (44, 45), these findings have not been demonstrated following single course regimens in preterm or late preterm infants (46, 47).

### 5.2 Intrauterine growth restriction (IUGR)

Various studies have demonstrated that premature infants born with IUGR have higher rates of BPD compared to gestation matched peers (48, 49). That adverse outcome reflects the underlying structural changes in the developing airways which have long term functional implications. Several observational studies assessing the lung function of school aged children born prematurely with IUGR have reported a reduction in forced expiratory volume in one second (FEV_1_) and an increase in airway resistance (50, 51). An association between early fetal growth restriction and asthma is well established in longitudinal birth cohort studies with follow up at 5, 10 and 15 years (52–54). More specifically regarding premature associated wheeze, a large cohort study demonstrated that a reduction in fetal growth between the first and second trimester was associated with a higher odds of “wheeze ever” outcome in childhood (OR: 1.59 95% CI: 1.01, 2.51) (55). Consistent with this, a recent retrospective study of 1,616 children investigating the risk factors associated with wheezing in preterm infants identified IUGR as contributing factor for the development of early wheeze (56). Animal studies have demonstrated asthma susceptibility following IUGR may be explained by structural-functional airway abnormalities such as airways smooth muscle (ASM) thickening and altered reactivity rather than by simple hypoplasia or size (57). The authors also suggest that IUGR is associated with an exaggerated inflammatory response (57). Both mechanisms are proposed as parallel contributors to an increased asthma risk. Future studies directly assessing airway structure and reactivity in IUGR-affected individuals are warranted.

### 5.3 Chorioamnionitis

Multiple systematic reviews have shown that histologic or clinical chorioamnionitis increases the risk of respiratory morbidity throughout the life course (58–60). A recent systematic review and meta-analysis, including eight studies and 35,000 infants, found that chorioamnionitis was significantly associated with wheeze/asthma in childhood (OR: 1.71, 95% CI: 1.55–1.89) (61). In a two years follow up study (Boston Birth Cohort), very preterm born children with a history of chorioamnionitis had the highest risk of wheezing (OR: 4.0, 95% CI: 2.0–8.0) and physician diagnosed asthma (OR: 4.4 95%CI: 2.2–8.7) (62). A retrospective cohort study of more than 500,000 singleton born children demonstrated that pregnancy complicated by chorioamnionitis was associated with increased incidence rates of childhood asthma with preterm born children more likely to be affected than term infants [incidence rate ratio, 2.9; 95% confidence interval (CI), 2.6–3.3] (63). The reported relationship between prematurity and chorioamnionitis was inversely correlated with gestational age even after adjustment for BPD. While the studies by Zhu et al. and Getahun et al. included a clinical diagnosis of chorioamnionitis, a small study of 115 children born at less than 34 weeks demonstrated histologically diagnosed chorioamnionitis was associated with a 2.72-fold increased risk of wheezing in children at 24–40 months (64). The mechanisms remain understudied but there is emerging evidence of airway smooth muscle (ASM) thickness and subsequent reactivity developing antenatally in response to perinatal inflammation conditions including preterm birth and chorioamnionitis (65).

### 5.4 Antenatal smoking

Antenatal exposure to tobacco smoke is a well-established risk factor for early wheeze and childhood asthma. The likelihood of developing asthma is increased by maternal smoking as well as exposure to second hand smoke throughout pregnancy (66–69). A prospective study of preterm infants found that maternal smoking approximately doubled the risk of BPD and was strongly associated with late respiratory morbidity in early childhood (70). Another cohort study reported an interaction between prematurity and antenatal smoking, where their combination markedly increased recurrent wheeze compared with either factor alone, suggesting synergistic effects on already vulnerable immature lungs (71). The mechanism underlying this predisposition involves the ability of nicotine to travel across the placenta causing vasoconstriction of vessels and impeding fetal lung development due to the lack of oxygen and nutrients (72, 73). Beyond structural lung development, prenatal tobacco exposure interferes with cellular differentiation and immune regulation promoting a Th2-skewed immune response and airway remodelling characterised by smooth muscle overgrowth, collagen deposition and heightened inflammatory reactivity, all of which contribute to an elevated risk of asthma and atopy in later childhood (74, 75). Furthermore, epigenetics also plays a role with maternal smoking inducing changes in DNA methylation patterns affecting gene expression. The AHRR gene which encodes for transcription involved in regulating immune responses has been implicated with a study showing that increased methylation of this gene was associated with asthma (76).

The evidence demonstrating that nicotine mediates the effects of antenatal maternal smoking on lung development suggests that nicotine replacement therapy (NRT) and e-cigarettes containing nicotine similarly affect lung development though the descriptive and mechanistic data describing these findings is limited (77). Studies of NRT and e-cigarette use in pregnancy have not identified adverse pregnancy outcomes regarding reduced birthweight or prematurity, but have not considered infant and childhood respiratory outcomes (78).

### 5.5 Breastfeeding

Multiple studies have demonstrated that breastfeeding has protective effects as it helps reduce the risk of wheezing and respiratory infections (79–81). A large prospective longitudinal study of 10,126 children analysing the impact of the length of breastfeeding on wheezing found that a longer duration of breastfeeding was associated with a lower likelihood of developing early transient wheeze (wheezing present up to five years of age) (82). A study analysing the impact of breastfeeding with recurrent wheeze among black preterm infants found that the number of episodes of wheezing were lower within the group of infants exclusively breastfed during their first three months of life (79). The protective benefits of breastfeeding persists beyond infancy as evidenced by a systematic review which reported an inverse relationship between breastfeeding and asthma risk in children up to two years of age (83). Such findings are consistent with the known immunogenic properties of breast milk which is composed of multiple components involved in the stimulation and development of the immune system (84). Amongst premature infants, lower rates of breastfeeding have been reported (85). Factors affecting post-partum breastfeeding differ based on the time interval post discharge with early factors more likely to be breast milk supply or technique while later factors more likely to be social including support and socio-economic factors (85). Socioeconomic status and lifestyle are important confounders in breastfeeding and socioeconomic factors can influence asthma development through multiple mechanisms other than breastfeeding which is discussed in a later section (86–88).

### 5.5 Antibiotics

Premature infants are commonly exposed to antibiotics in the neonatal period. Whilst antibiotic treatment is clinically necessary, growing evidence suggests that early and prolonged antibiotic exposure may have unintended consequences for respiratory health, primarily through disruption of the developing infant microbiome and subsequent influence on immune development. The early postnatal period, particularly the initial six months of life, represents a critical window for gut microbial development (89). Administration of antibiotics causes a reduction in the microbial diversity resulting in abnormal expansion of immune cells, higher proportion of pro-inflammatory cytokines and disrupted immune regulation (90). A systematic review evaluating the long term outcomes of prenatal antibiotic exposure in children found an increased risk of wheezing (OR: 1.39, 95% CI: 1.14–1.69, *p* < 0.01) and asthma (OR: 1.36, 95% CI: 1.24–1.50, *p* < 0.01) (91). These associations were similarly observed in premature infants (92). Evidence suggests that the impact of early antibiotic exposure extends into childhood with a study identifying an increased likelihood of asthma at five years of age among children who had received antibiotics in their first year of life (93). Establishing causality, however, remains challenging as the indication for antibiotic treatment, namely infection, may itself be a risk factor for subsequent respiratory morbidity.

### 5.6 Early childhood viral illness

Respiratory infections are widely recognised as a risk factor linked to the development and persistence of premature associated wheeze. Respiratory syncytial virus (RSV) is one of the most common viral pathogens to which preterm infants are particularly susceptible. Multiple studies have demonstrated that infants born before 32 weeks of gestation frequently require hospital admission for respiratory diseases, predominantly of infectious aetiology, within their first two years of life (94, 95). In the MAKI study severe RSV infection was identified as a key factor contributing to the development of recurrent wheeze during the first year of life in premature infants (96). The subsequent SPRING study showed that premature infants who had been hospitalised for RSV were more likely to develop recurrent wheeze up to six years of age compared with those who were not hospitalised (46.7% vs. 27.4%; *p* = 0.001) (97). Furthermore, a systematic review evaluating the impact of RSV infection on respiratory morbidity found that RSV infection in infancy is closely linked to recurrent wheezing and asthma that extends into childhood (98). The exact mechanism underpinning those observed trends is not fully understood however, several theories have been proposed. These include epithelial damage and structural changes resulting from persistent airway inflammation, chronic stimulation of atypical inflammatory pathways and dysregulation of immunomodulatory cytokines, namely IL-10, all of which lead to prolonged airway hyperresponsiveness (99–103). Taken together, the evidence strongly supports that RSV infection in early life confers a substantial and prolonged risk of wheeze and asthma in premature infants.

Severity of RSV illness may predispose to later wheeze. PICU admission has been associated with a nine-fold higher odds of wheezing in survey data (104). Infants mechanically ventilated for RSV show high rates of small airway dysfunction and ventilation inhomogeneity on follow-up testing, though prematurity was not itself an independent risk factor in this cohort (105). Whether this results in higher incidence of childhood wheeze or asthma needs investigating.

Palivizumab is an anti-RSV monoclonal antibody for preventing RSV infections. Palivizumab prophylaxis in preterm born infants resulted in a reduction in wheezing days, recurrent wheeze and use of bronchodilators (96, 106–109). Long term follow up has not demonstrated a major effect on lung function or the prevalence of physician-diagnosed asthma at six years of age (110, 111). Clinical trials of Nirsevimab, a single dose anti-RSV monoclonal antibody, have demonstrated promising results including reduced RSV related lower respiratory tract infections, hospitalisation and severe RSV cases (112, 113). National programmes of Nirsevimab prophylaxis (France, Spain, Ireland and Luxemburg) have demonstrated marked reductions in RSV-related hospitalisations and intensive care admissions (97, 114–117). Follow-up studies are needed to investigate if this will result in a wheeze or asthma risk reduction or improvement in lung function.

Prenatal infection with RSV may result in vertical transmission and adverse neonatal respiratory outcomes (118, 119). Cord blood analysis of infants born to mothers with prenatal RSV infection has demonstrated virus specific cytokine and chemokine-mediated inflammatory responses even in the absence of viral particles. Trinh et al. have demonstrated a significant correlation between viral load and inflammatory mediators with birth weight and postnatal weight gain (118). These findings suggest that virus-induced perinatal inflammation may alter postnatal development and metabolism and may constitute an early origin of pathological outcomes presenting later in childhood. Maternal RSV infection may also predispose offspring to postnatal lower airways dysfunction and ASM contraction during early-life RSV reinfections. Maternal RSV vaccination schedules have had a significant impact on reducing RSV-related lower respiratory tract infections (LRTIs) requiring medical attendance. This passive immunity helps shield infants from severe RSV-related LRTIs during their first 6 months of life (115). For preterm infants, who are at greater risk of severe RSV infection, maternal vaccination reduced infants risk of hospitalisation by 69.4% when mothers were vaccinated at least 14 days before birth (120). It, however, has not been established whether these interventions will reduce the risk of subsequent wheezing episodes or prevent asthma development.

Human rhinovirus (HRV) respiratory infections have also been associated with higher risk of asthma in childhood with prematurity notable as an independent factor associated with asthma at 6–9 years (121). A recent meta-analysis suggests that HRV-wheezing illness in the first three years is associated with subsequent wheezing and asthma and this association remains significant at follow up in children greater than 10 years of age (122). Focusing on preterm infants, HRV-infected infants suffer greater chronic respiratory morbidity and health care related costs compared with no LRTI and RSV-LRTI infected infants. In follow up studies of more than 150 infants born less than 36 weeks of gestational age, at one year infants with a history of a HRV-LRTI had more outpatient and respiratory-related general practitioner attendances and more wheeze than the no LRTI group and more respiratory-related outpatient attendances than the RSV-LRTI group (123). Of the subtypes of HRV, infants who had HRV-C LRTI were more likely to wheeze and use respiratory medications than the no LRTI group (124). In the same infants, healthcare related costs were calculated from the National Health Service reference costing scheme and healthcare utilisation determined by examining hospital/general practitioner records (123). Compared with the no LRTI, combined RV/RSV-LRTI had the greatest increase in adjusted mean cost (difference GBP 5769), followed by the RV LRTI group (difference GBP 278) and, finally, the RSV LRTI group (difference GBP 172) (*p* = 0.045) (123).

Preterm infants are predisposed to HRV-LRTI by reduced neonatal lung function and a genetic predisposition (125). In a study of infants born at less than 36 weeks gestational age, infants that developed HRV-LRTIs by one year had reduced compliance (1.6 vs. 1.2 mL/cmH2O/kg) at 36 weeks postmenstrual age (125). In addition, a single nucleotide polymorphism in the gene coding for the vitamin D receptor was associated with the development of HRV LRTIs and any viral LRTIs (125).

### 5.7 Gastro oesophageal reflux

Gastro oesophageal reflux (GOR) is a common phenomenon in infancy and children (126) with the prevalence of gastro-oesophageal reflux disease (GORD) varying across populations (127–129). The primary mechanism for GOR in infants is transient lower oesophageal sphincter relaxation (130). Preterm infants appear to be more commonly affected (131); a history of BPD putting infants particularly at risk, although the evidence is conflicting (132, 133). Cohort studies have reported GOR prevalence estimates of up to 42% in infants with BPD (134). Preschool children frequently present with extra-oesophageal symptoms such as wheeze and cough (126, 135), which may be misattributed to primary respiratory diagnoses such as viral-induced wheeze or poorly controlled asthma (136). Symptom management in this cohort remains a challenge. In a prospective cohort study of children aged greater than one year who underwent pH-impedance monitoring, Zenzeri et al. demonstrated children with typical gastrointestinal symptoms (pyrosis, regurgitation) exhibited predominantly acidic reflux while children presenting with respiratory symptoms demonstrated a significantly higher frequency of weakly alkaline and non-acidic reflux episodes (mean 22.1 vs. 10.1) (137). Those findings support the hypothesis that respiratory symptoms are less related to acidity than GI symptoms and may account for the low efﬁcacy of acid suppressive treatment reported in patients with respiratory symptoms. There is emerging evidence of an association between GORD and later development of asthma in childhood (138). A recent longitudinal study tracking over 85,000 children demonstrated that a diagnosis of GORD in the first year of life was an independent risk factor for subsequent development of asthma (6.5% vs. 3.7% in controls) (139). The cohort included infants born at less than 36 weeks of gestation, although subgroup analysis on the preterm infants was not performed.

### 5.8 Pollution

Exposure to outdoor and indoor pollution during pregnancy and early life appears to worsen respiratory health and increase the risk of asthma with premature infants having heightened vulnerability. Exposure to common air pollutants such as particulate matter (PM) and nitrogen oxides (NOx) during the neonatal period can lead to impairment of postnatal lung function that can persist into early childhood (140). A prospective cohort study investigating the effect of common ambient air pollutants on lung function using spirometry on school aged children who were born prematurely found that exposure to PM_2.5_ and nitrogen dioxide was associated with a reduction in forced vital capacity (FVC) (141). A systematic review, which included 26 studies, concluded that prenatal exposure to PM and NOx was linked to a higher likelihood of developing asthma with the most critical period being during the second trimester (142). Similarly, wheeze has been shown to occur at a higher frequency and earlier onset in preterm infants with greater pollution exposure, particularly in urban settings (143)^.^ Those observed trends can be attributed to pollution causing airway inflammation, oxidative stress and disrupted alveolarisation affecting the structure of the premature lung (144). The presence of indoor allergens (mould, pet allergens), activities such as smoking, heating and cooking and the adequacy of indoor ventilation all contribute to wheeze and asthma risk (145). Increased levels of PM_10_ and PM_2.5_ in the indoor environment have been linked to an increased burden of respiratory symptoms, increased use of asthma medications, more severe exacerbations and hospital visits among children with an asthma diagnosis (146, 147). While air filters may reduce exposure to fine particles, their impact on symptoms or lung function remains elusive (148).

### 5.9 Microbiome

The airway and gut microbiota of premature infants are shaped by early life interventions resulting in disturbances in their composition and diversity which has been linked to adverse respiratory outcomes. A prospective study of infants born at less than 32 weeks of gestation demonstrated that the microbial profile of the nasopharyngeal airway and gut during the first week of life could predict the risk of recurrent wheeze in the first year, with those who developed wheeze exhibiting a pathogen community predominant *in Klebsiella, Escherichia/Shigella* and *Stenotrophomonas* (149). Factors potentially mediating this observed early dysbiosis include antibiotic exposure, feeding using artificial formula and use of ventilatory support (149). Further observational studies have shown that early colonisation of the airway microbiota with *Staphylococcus, Moraxella* and *Oxalobacteraceae* is associated with a greater risk of wheezing potentially driven by impaired immune responses resulting in a more pro-inflammatory state (150–152). Conversely, early prevalence of commensal such as *Bifidobacterium* and *Staphylococcus* appeared to confer a protective effect likely through immunomodulatory mechanisms (153). While the current evidence provides an insight into the association between the microbiome and premature associated wheeze, it remains unclear whether microbial imbalance is a direct causal factor of wheeze or a marker of an underlying susceptibility.

### 5.10 Socioeconomic

Health inequality is increasingly becoming recognised as an important factor which can affect lung health. A prospective longitudinal cohort study demonstrated that maternal educational level influences childhood wheezing, with mothers without a formal academic qualification having children at increased risk of developing wheeze in early infancy compared to mothers with a degree (154). This observed pattern may potentially be mediated by maternal behaviours linked to education, particularly smoking during pregnancy and attitude to breastfeeding. Furthermore, analysis of the same cohort showed that children from lower income households were more likely to experience adverse respiratory health, including wheezing (155). In addition to socioeconomic factors, race and ethnicity are linked to respiratory morbidity. In an observational study of a cohort of 5,809 children, there was a higher incidence of early and persistent wheeze amongst the Black and Hispanic children (100). Collectively, those findings emphasise the importance of considering the effects of social determinants of health on long term respiratory outcomes.

## 6 Lung function trajectories

Preterm born individuals, as compared to term born populations, are at increased risk of abnormal trajectories of lung function and early symptomatic respiratory disease (34, 156). Comprehensive aggregate data on long-term lung function following preterm birth have recently been reported (157–159). In a meta-analysis of studies across all ages with term-born controls including 5,501 preterm and 12,648 term individuals, preterm individuals had lower FEV_1_ [standard mean difference (SMD) [95% CI]: −0.67 [−0.75 to −0.58]] and lower FEV_1_/FVC [SMD [95% CI]: −0.56 [−0.68 to −0.45]] compared to term-born controls (159). Preterm infants with a history of BPD vs. those without BPD demonstrated a more severe decline in FEV_1_ (SMD [95% CI] −0.67 [−0.78 to −0.57) and FEV_1_/FVC [SMD [95% CI] −0.38 [−0.50 to −0.25]] (159). Those results are consistent with previously reported data on expiratory airflow limitations throughout the lifespan in preterm cohorts (157, 160). Early-life wheeze predicts poor lung function trajectories but its effect on the lung function trajectories and respiratory symptom burden in preterm born individuals is understudied (161). In a retrospective analysis of three longitudinal birth cohorts (162–164), [the Manchester Asthma and Allergy Study (MAAS), a UK-based cohort designed to investigate the development of asthma and allergic disease; the Avon Longitudinal Study of Parents and Children (ALSPAC), a large population-based UK cohort following children born in the early 1990s; and a population-based Australian birth cohort with follow-up into early adulthood], a persistently low trajectory for FEV_1_ was associated with wheeze and asthma throughout follow-up (162–164). Notably, prematurity was only examined in one of these cohorts (164).

Asthma can be associated with lung function decline and those with poorly controlled asthma worse affected (165). In a Danish population study (the Copenhagen City Heart Study) FEV_1_ measurements were made over 15 years in 17,506 subjects, including 1,095 with asthma. Those with asthma showed a decline in FEV_1_ of −38 mL/year compared with −22 mL/year in healthy individuals (166). In a UK population study (Optimum Patient Care Research Database) for each additional exacerbation there was a loss of peak flow of −1.34 litres/minute (95% CI −1.23 to −1.50) in measurements made over 10 years (167). Patients with an acute exacerbation rate (AER) of greater than two lost an additional −39.3 mL FEV_1_ per year compared with patients with no exacerbations (95% CI −65.2 to −13.4; *p* = 0.008). The authors, however, did not examine the effect on premature born individuals (167).

The mechanisms linking asthma exacerbations to accelerated functional decline were first proposed by Bai et al., who suggested that repeated periods of intense airway inflammation play a central role (168). In a historical cohort study of 93 non-smoking patients with moderate-to-severe asthma, they demonstrated an association between exacerbation frequency and a more rapid decline in lung function. They hypothesised that the effect resulted from recurrent episodes of heightened airway inflammation (168). The study was conducted prior to the widespread use of inhaled corticosteroids, which minimises potential treatment-related confounding.

Longitudinal studies of the natural history of asthma support that impaired lung function in adult asthmatics often has its origins in childhood (169, 170). About one-third of participants in the Dunedin (New Zealand) birth cohort who had persistent wheezing and airway hyperresponsiveness in childhood developed signs of fixed airflow limitation by adolescence (170). This was shown by a post-bronchodilator FEV_1_/FVC ratio that was more than two standard deviations below the average for their age- and sex-matched, non-asthmatic, non-smoking peers. As they moved into adulthood, their lung function became even less reversible. These fixed changes were more noticeable in those who had received inhaled corticosteroids, though this likely reflects that they had more severe disease rather than an effect of the medication itself.

Lung function impairment in childhood asthmatics may indicate more severe forms of disease progression and place these children at increased risk of fixed airflow obstruction in early adulthood. In an observational follow-up study over 13 years, 684 study participants had lung function assessed at three time points from enrolment at the age of 5 to 12 years with the last spirometric measurement in the third decade [mean age ( ± standard deviation) 26.0 ± 1.8 years] (171). Patterns of lung function were defined as normal growth, early decline, reduced growth, reduced growth and early decline. These are consistent with GOLD spirometric criteria for lung-function impairment. The authors reported 514 (75%) of participants had abnormal patterns of lung function with over half [256 (52%)] of participants demonstrating early decline. Additional risk factors were premature birth and childhood respiratory infections.

Simpson et al. assessed a cohort of 200 preterm (less than 32 weeks of gestation) children at 4–8 years and 9–12 years. The lung function (FEV_1_, FEV_1_/FVC, forced expiratory flow between 25% and 75% of vital capacity (FEF_25–75_), and respiratory system reactance at 8 Hz of children reporting wheeze declined more than 0.5 zscores in children who had never wheezed (156). In a review of paediatric patients from the Center for Applied Genomics biobank (USA), patients with asthma and at least five years of follow up data were selected with 4% of the cohort born at less than 32 weeks of gestation. Regression models demonstrated that those individuals reported 20.5% more asthma exacerbations than a term born control group (95% CI: 0.6%–44%; p. = 0.04) (172). In the same study, patients born less than 32 weeks of gestation had a 61% higher likelihood of experiencing exacerbations past the age of six emphasising that prematurity is not only a risk factor for early asthma morbidity but also for sustained respiratory problems into later childhood and potentially adolescence (172).

## 7 Prediction of respiratory outcomes

Various definitions of BPD have been used but often do not adequately predict childhood morbidity (173). Eighteen definitions were compared in 2,677 infants born less than 32 weeks of gestation from 18 US centres with regard to their ability to predict death or serious respiratory morbidity through 18 to 26 months of age (173). The best predictor of outcome was the mode of respiratory support administered at 36 weeks postmenstrual age regardless of supplemental oxygen use. An alternative approach to prediction is cluster analysis. Premature infants could be classified into three groups by hierarchical agglomerative cluster analysis using readily available clinical data that is birthweight and duration of mechanical ventilation (173). Individuals in clusters two and three required prolonged mechanical ventilation (at least six days) and cluster three had birthweights less than 882 gms (174). Compared to cluster one, clusters two and three had significantly more LRTIs; cluster two RSV LRTIs and cluster three rhinovirus LRTIs. Use of such a tool may help to identify infants at greater risk of respiratory morbidity and requirement for more intense follow-up.

## 8 Management: why asthma pathways may not be appropriate in premature individuals

The GINA Strategy Report provides clinicians with an annually updated evidence-based strategy for asthma management and prevention (16). These are considered the international gold standard of asthma management. It recommends a stepwise approach to treatment with a preference for inhaled corticosteroid (ICS) containing regimens including maintenance and reliever therapy (MART) (175, 176). Inhaled corticosteroids are the cornerstone of asthma management with long-acting beta_2_-agonists (LABAs) used as add-on therapy in combination. Inhaled corticosteroids primarily exert their effects through glucocorticoid receptor mediated modulation of airway inflammation reducing proinflammatory gene expression, inflammatory cell recruitment and a reversal of capillary permeability (177). A Cochrane review has demonstrated the effectiveness of regular ICS vs. placebo in chronic asthma in both children and adults (178). While ICS are effective in improving outcomes in eosinophilic asthma phenotypes (179, 180) they are less effective in patients with neutrophilic airway inflammation which is associated with a poorer response to corticosteroid therapy (181). The applicability of ICS in the management of wheeze and asthma in individuals born preterm remains uncertain. Those populations typically exhibit lower FeNO levels and reduced blood eosinophil counts suggesting a lower prevalence of eosinophilic inflammatory phenotypes, while neutrophilic airway inflammation has been described (7, 30, 182).

Long-acting β_2_-agonists (LABAs) act via relaxation of ASM. Cochrane reviews suggest that in children with persistent asthma, the addition of a LABA to inhaled corticosteroids improves lung function and symptom control but does not significantly reduce exacerbations compared with higher-dose ICS alone, and LABAs should not be used without concomitant ICS due to safety concerns (183, 184). Evidence of ICS and LABA in children born preterm is limited. While some individuals demonstrate bronchodilator responsiveness their role remains uncertain. In a narrative review, Course et al. summarised the evidence from trials of ICS in preterm born children (185). Four studies were described, with a total of 285 patients and five treatment regimens highlighting the heterogeneity of the studies' methodology (186–189). The conducting airways in preterm born, particularly those with BPD, may show ASM remodelling which is typical in children with chronic inflammatory lung disease such as asthma (190, 191). *In vivo* studies have demonstrated a positive relationship between the volume fraction of ASM and bronchodilator responsiveness in children with asthma (191). Whether this may result in a greater bronchodilator responsiveness in the preterm lung with asthma has yet to be reported.

Long-acting muscarinic antagonists (LAMA) may be used as an add-on therapy. The mechanism of action involves the blocking of M3 muscarinic receptors on ASM, inhibiting acetylcholine-induced bronchoconstriction (192). A systematic review of three studies demonstrated LAMA use as add-on in moderate or severe asthma in adolescents (aged 12–17 years) improved lung function (peak FEV_1_) compared to a placebo control group who were continued on maintenance therapy (193). In younger children (aged 6–11 years), a recent systematic review of four RCTs and two observational studies including 1,210 participants demonstrated that LAMA significantly improved peak FEV_1_ (mean difference 86.16 mL; 95%CI: 18.62–153.71) and FEF_25%–75%_ (mean difference 0.2518L; 95%CI: 0.1971–0.3064) (194). Asthma control measured by the Asthma Control Questionnaire also favoured treatment with LAMA. No studies have evaluated the effectiveness of LAMAs in preterm born individuals (195).

More recently, two randomised controlled double-blind placebo-controlled trials have demonstrated some improvement in lung function of preterm born children treatment with ICS regimens. The PICSI trial assessed whether regular fluticasone propionate improved lung function in a 12 week period in children who had been born very preterm (less than 32 weeks of gestation) (189). A total of 170 children were randomly assigned with a mean increase of 0.3 FEV_1_ z-score (∼4% predicted) in the preterm group, which is unlikely to be clinically significant. The RHiNO trial evaluated preterm infants born at less than 34 weeks of gestation with pre-trial %FEV_1_ less than or equal to 85% and included 53 children, 20 receiving ICS, 19 receiving ICS/LABA and 14 receiving placebo. The combination therapy (ICS/LABA) was associated with a significant increase in % FEV_1_ of 14.1% (95% CI: 7.3–21.0; *p* = 0.002) compared with placebo (188). Among steroid-naïve participants, the combination therapy led to FEV_1_ improvements of 10.2% (95% CI: 3.8–16.5; *p* = 0.03) relative to corticosteroids alone and 17.2% (95% CI: 10.2–24.2; *p* ≤ 0.001) relative to placebo. By comparison, corticosteroids alone produced a non-significant improvement of 7.0% (95% CI: −0.9 to 15.0; *p* = 0.26).

## 9 Management of prematurity-associated lung disease with wheeze: treatable traits

A treatable-traits framework offers a more useful approach to wheeze management in preterm-born children than a standard asthma pathway (Table 1). Rather than viewing “asthma” as a single disease, clinicians identify clinically relevant, measurable and modifiable traits, including airway phenotype, comorbidities, environmental exposures and self-management factors and target treatment accordingly. This shifts the focus from “does this child have asthma?” to “which treatable traits are present, and what evidence supports intervention?”

**Table 1:** Investigations in preterm-born children with wheeze—treatable trait .

Domain	Investigation	Trait identified	Considerations in preterm-born children
Airflow obstruction	Spirometry (FEV0.5, FEV_1_, FVC, FEV_1_/FVC, FEF_25–75_) with bronchodilator reversibility	Fixed versus variable airflow obstruction Reversible component	Feasible from approximately five years of age. Lower baseline FEV_1_ expected; Absence of reversibility does not exclude a treatable obstructive component.
	Impulse oscillometry	Small airway function; peripheral airway resistance	Useful in younger children unable to perform spirometry. Tidal-breathing technique requires minimal cooperation.
	Multiple-breath washout (lung clearance index)	Ventilation inhomogeneity	
Lung volumes and gas transfer	Body plethysmography Gas transfer	Hyperinflation; gas trapping Impaired alveolar gas exchange	DLCO is frequently reduced in BPD Can be useful in distinguishing prematurity-associated lung disease from classical asthma.
	Cardiopulmonary exercise testing	Functional exercise capacity	Recommended from approximately eight years of age. CPET helps quantify functional limitation,
Airway inflammation	Fractional exhaled nitric oxide (FeNO)	Eosinophilic (T2-high) airway inflammation	Often low in preterm-born wheezers, supporting a low-T2 phenotype. FeNO decreases with inhaled corticosteroid treatment in this subgroup, suggesting it may help identify steroid-responders. A normal FeNO does not exclude clinically relevant disease.
	Blood eosinophil count, total IgE, specific IgE or skin prick testing	Atopic status; T2-high biomarkers	Helps distinguish coexistent atopic asthma from prematurity-associated lung disease and informs likely response to inhaled corticosteroids.
	Induced sputum cell counts	Neutrophilic, eosinophilic, mixed or paucigranulocytic phenotype	Neutrophilic phenotype in some preterm-born cohorts
Structural lung disease	High-resolution CT chest/Chest MRI	Mosaic attenuation, small airway disease, Hypodense areas (emphysema, bullae, cysts); hyperdense areas (atelectasis, consolidations, subpleural opacities, bronchial wall thickening); architectural distortion; tracheobronchomalacia; subglottic stenosis	Reserve for atypical course, treatment failure, severe symptoms or unexplained exercise limitation, Balance against radiation exposure. Routine chest imaging is not recommended
Pulmonary vascular and cardiac	Echocardiography Cardiac MRI; chest CT with contrast;	Pulmonary hypertension; right ventricular function; structural cardiac disease Pulmonary vein stenosis; cardiac anatomy and function; pulmonary blood flow	Recommended in infants with severe BPD
Extra-pulmonary treatable traits	pH/impedance study; Videofluoroscopic swallow assessment	Gastro oesophageal reflux disease; Aspiration; unsafe swallowing; feeding difficulties	Threshold for investigation should be low when symptoms are atypical or refractory.
	Polysomnography or respiratory polygraphy or overnight oximetry	Obstructive sleep apnoea; nocturnal hypoxaemia	Consider in children with disturbed sleep, daytime somnolence or unexplained pulmonary hypertension.
	ENT review ± bronchoscopy	Upper airway contribution; subglottic stenosis; vocal cord dysfunction	Consider in children with prolonged intubation, persistent stridor, or recurrent croup or daily barking cough

For children with reversible airway obstruction, a 6–8 week trial of ICS with or without a LABA is reasonable. The RHiNO trial demonstrated a 14.1% improvement in % predicted FEV_1_ with ICS/LABA over 12 weeks, greater than ICS alone, while the PICSI trial showed a smaller but measurable benefit from ICS monotherapy in a responsive subgroup. Response should be reviewed objectively after 6–8 weeks using lung function and FeNO, where possible, and treatment continued only if clinically beneficial.

LAMAs, such as tiotropium, reduce cholinergic bronchoconstriction and are licensed as add-on therapy for poorly controlled paediatric asthma from six years of age. There are no randomised trials in preterm-born children, so their role remains uncertain. Pragmatically, LAMAs may be considered in older children with persistent asthma symptoms despite ICS/LABA, particularly where bronchodilator responsiveness is demonstrated, with objective reassessment after 6–8 weeks.

Where imaging demonstrates structural lung disease, including bronchiectasis or bronchial wall thickening, airway clearance techniques, nebulised saline or hypertonic saline, and prompt antibiotic treatment during exacerbations may be more appropriate than escalating inhaled steroids. Recurrent infections are themselves a treatable trait: routine immunisations should be optimised, including influenza vaccination and RSV prevention through maternal vaccination and Nirsevimab prophylaxis.

Extrapulmonary contributors are common and often drive respiratory symptoms. Gastro-oesophageal reflux, aspiration and swallowing dysfunction may require speech and language assessment, videofluoroscopy or pH impedance studies. Feeding modification or anti-reflux measures proving more beneficial than additional inhalers. Sleep-disordered breathing should also be considered early, with overnight oximetry or respiratory polygraphy or polysomnography where indicated. Upper airway abnormalities such as laryngomalacia, tracheomalacia, bronchomalacia, vocal cord dysfunction or subglottic stenosis are common after prolonged ventilation and may warrant ENT assessment and bronchoscopy. Cardiac sequelae, including pulmonary hypertension, justify echocardiographic evaluation in severe cases. Growth, nutrition and bone health should also be optimised, given their impact on long-term lung function.

Behavioural and environmental factors are frequently overlooked but highly modifiable. Inhaler technique and adherence should be reviewed at every consultation before treatment escalation, as many cases of apparent treatment failure reflect poor adherence or device misuse. Tobacco smoke exposure, vaping and air pollution should be addressed routinely, with smoking cessation support offered where relevant. Exercise capacity is often reduced in preterm-born children, but structured exercise programmes can improve fitness and quality of life. Psychological support may also be required to address barriers to adherence and physical activity, especially during adolescence.

## 10 Future directions

Despite the heavy burden of chronic respiratory morbidity in prematurely born individuals, an international survey demonstrated that the respiratory follow-up of even those born extremely prematurely is not standardised, particularly after the first two years after birth (196). Practitioners surveyed stated that this reflects there are no widespread guidelines and that healthcare professionals and even the individuals and their families themselves may be unaware of the long-term respiratory outcomes of premature birth. A consensus from neonatologists, paediatricians, allergologists and adult chest physicians was that more education was required about this for individuals, their families and practitioners. There should be better integration of medical records and affected individuals should carry a card demonstrating key factors in their medical history to present at all interactions with healthcare professionals. Furthermore, particularly extremely prematurely born individuals should have lifelong respiratory follow-up so that they don't just “pop up” when they become symptomatic. To ensure the highest risk individuals are followed up, not just those born extremely prematurely, more prediction studies are required and modifiable risk factors identified. It is essential that personalised treatment is delivered and that it is recognised wheeze may not be asthma.

## 11 Conclusion

Preterm born infants are at increased risk of respiratory morbidity including recurrent wheeze. As a consequence, affected individuals are often given an incorrect diagnosis of asthma. Understanding the pathophysiology of wheeze in these infants and identifying modifiable or treatable traits are important for the correct diagnosis and precise management of the symptoms. Future studies should focus on clinical studies of pharmacological and non-pharmacological therapies to improve symptom burden and long-term outcomes.
